# Mirrors in the PDB: left-handed α-turns guide design with D-amino acids

**DOI:** 10.1186/1472-6807-9-61

**Published:** 2009-09-22

**Authors:** Srinivas Annavarapu, Vikas Nanda

**Affiliations:** 1 Department of Biochemistry, Robert Wood Johnson Medical School, University of Medicine and Dentistry of New Jersey, Piscataway, NJ 08854, USA; 2 Center for Advanced Biotechnology and Medicine, Piscataway, NJ 08854, USA

## Abstract

**Background:**

Incorporating variable amino acid stereochemistry in molecular design has the potential to improve existing protein stability and create new topologies inaccessible to homochiral molecules. The Protein Data Bank has been a reliable, rich source of information on molecular interactions and their role in protein stability and structure. D-amino acids rarely occur naturally, making it difficult to infer general rules for how they would be tolerated in proteins through an analysis of existing protein structures. However, protein elements containing short left-handed turns and helices turn out to contain useful information. Molecular mechanisms used in proteins to stabilize left-handed elements by L-amino acids are structurally enantiomeric to potential synthetic strategies for stabilizing right-handed elements with D-amino acids.

**Results:**

Propensities for amino acids to occur in contiguous α_L _helices correlate with published thermodynamic scales for incorporation of D-amino acids into α_R _helices. Two backbone rules for terminating a left-handed helix are found: an α_R _conformation is disfavored at the amino terminus, and a β_R _conformation is disfavored at the carboxy terminus. Helix capping sidechain-backbone interactions are found which are unique to α_L _helices including an elevated propensity for L-Asn, and L-Thr at the amino terminus and L-Gln, L-Thr and L-Ser at the carboxy terminus.

**Conclusion:**

By examining left-handed α-turns containing L-amino acids, new interaction motifs for incorporating D-amino acids into right-handed α-helices are identified. These will provide a basis for *de novo *design of novel heterochiral protein folds.

## Background

Solid phase chemical synthesis allows for the incorporation of non-natural amino acids into polypeptides[1]. The field has developed rapidly, permitting the construction of synthetic, protein-sized molecules. This has allowed protein chemists to explore the physical and biological effects of varying amino acid stereochemistry. A dramatic example was the chemical synthesis of the ninety-nine amino acid long HIV-1 protease from both L and D-amino acids[2]. The resulting enantiomeric molecules were both well-folded and specifically active on a protease substrate of the same respective amino acid chirality as the enzyme. In this study, we use the Protein Data Base (PDB) as a source of structural information for specific D-amino acid sidechain interactions with α-helical backbones.

Much of the work on the role of variable stereochemistry on structure and stability has been conducted on short peptides [3,4]. This work has been motivated by natural examples of polypeptides that combine L and D amino acids. The antimicrobial toxin, gramicidin, is a well studied example of such a molecule, containing alternating L and D amino acids. This allows it to adopt the β-helix, a novel secondary structure composed of alternating positions in the β_L _and β_R _conformation[5,6]. The β-helix has been used as the foundation for novel cyclic peptide folds[7] and peptide nanotubes with ion channel activity and antimicrobial properties [8-11]. Other microbial peptides such as tolaasin use D-amino acids to enforce sharp bends in an α-helical domain [12]. Methods are being developed for incorporating L and D amino acids in computational *de novo *protein design [13-16].

Another practical application is the development of thermostable proteins that incorporate D-amino acids. Amino acids in proteins are rarely found in backbone conformations with positive φ and ψ angles at the α_L _region of Ramachandran space [17]. This paucity of α_L _residues is primarily due to unfavorable interactions between the sidechain and its backbone carbonyl and that of the preceding residue. The energetic cost of this steric clash has been estimated at around 1 kcal/mole by replacing L-Ala with D-Ala in a model α_R_-helical peptide [18,19]. The only amino acid that does not contribute this type of steric clash is Gly, which lacks a sidechain. Consequently, α_L _positions in proteins are primarily occupied by Gly [20,21]. This feature of glycine has been applied to the thermostabilization of a bacterial formate dehydrogenase which has five non-glycine amino acids throughout the protein in the α_L _conformation. Replacing these amino acids with Gly increases the activity at otherwise inactivating temperatures[22].

The backbone amide of glycine makes hydrogen bonding with exposed carbonyls at the C-terminal end of a helix [23-26]. This allows the chain to maintain a network of stabilizing interactions while terminating the helix and changing the direction of the chain. Other amino acids besides glycine are sometimes found in such positions, but are rare due to steric constraints already mentioned. Small polar amino acids are commonly found at the N-terminus of an α_R _helix, making sidechain hydrogen bonds to exposed amides of the backbone [27-32]. Together, these interactions are called 'helix caps'.

D-amino acids can function as C-terminal helix caps. While substitution of α_L _positions with Gly may remove unfavorable contacts, the entropic cost of fixing glycine in a given conformation can mitigate energetic benefits gained. D-amino acids, which favor the α_L _conformation, have been substituted for Gly, sometimes resulting in increased protein stability [33-35]. Observed folding free energy changes have ranged from zero to over two kcals/mol. In a monomeric helical peptide, adding D-Ala to the C-terminus of a helix resulted in no significant change in stability whereas D-Arg increased stability by approximately one kcal/mole, presumably due to stabilization of the helix macrodipole [36]. These varying results indicate that the roles of sidechain identity and stereochemistry in protein stability are still an open problem.

While much has been learned about standard capping interactions from the analysis of high-resolution protein structures in the PDB, the number of proteins containing D-amino acids is very low. Approximately 150 entries in the PDB contain D-amino acids that are not artifactual, and most of these are shorter than twenty amino acids[32]. A handful of these contain D-amino acids in helix C-capping contexts [1,34]. A number of designed heterochiral peptides are in the Cambridge Structural Database (CSD) of small molecules, but these are of limited use for the unbiased discovery of novel capping interactions.

One possible source of information is a set of small, contiguous *left*-handed turns and helices in proteins. These are rare due to the unfavorable steric interactions required to place L-amino acids in the α_L _conformation. For cases where such structures do exist, they often play key structural and functional roles [37]. Stabilizing interactions identified in a study of naturally occurring left-handed structures would be perpetrated by L-amino acids. Hence, the value to protein engineering and design is to realize that the structural enantiomer of such interactions would involve right-handed structures stabilized with D-amino acids.

This report outlines the search of a non-redundant subset of the PDB for left-handed turns and short α_L _helices. The total fraction of amino acids in the α_L _conformation is 4%, over half of which is attributed to glycine [20]. Despite this, a small set of left handed structures are identified for structural analysis. The intrinsic α_L_-helical preferences of most amino acids correlate with thermodynamic scales for inserting D-amino acids into α_R _helices. Furthermore, several N- and C-terminal capping motifs unique to left-handed helices are described. These are tantalizing candidates for novel D-amino acid capping motifs of α_R_-helices. Implications for protein stabilization and heterochiral protein design are discussed.

## Results and Discussion

### Backbone Geometry in Left-handed Turns and Helices

A non-redundant subset of structures in the PDB was searched for three or more contiguous residues in an α_L _conformation. Seventy-two three-residue turns, ten four-residue helices and two five-residue helices were found (see Additional Files 1, Table S1). In order to keep nomenclature consistent with previous studies [25], the relative positions of amino acids within these turns and helices are described as follows: the Ncap residue is the first amino acid in a contiguous left-handed conformation; the Ccap residue is the last amino acid in a contiguous left-handed conformation. The remaining positions are described in their position relative to the Ncap or Ccap:

---N"'-N"-N'-Ncap-N1-N2-N3....

   ...C3-C2-C1-Ccap-C'-C"-C"'

In three residue turns, N1 = C1.

**Figure 1:**
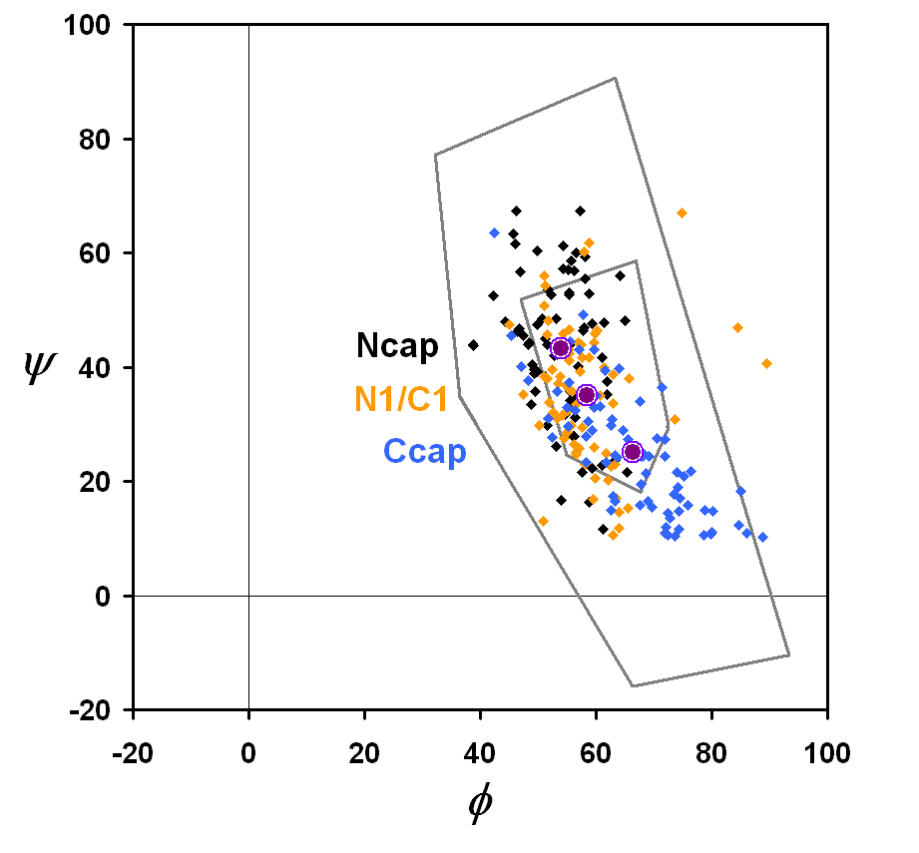
**Ramachandran plot of three-residue left-handed turns**. Plot of *φ *versus *ψ *values for residues at the Ncap (black), N1/C1 (orange) and Ccap (blue) positions. Means and standard deviations of (*φ*, *ψ*) (purple) for Ncap, N1/C1 and Ccap are (54 ± 6°, 43 ± 13°), (58 ± 7°, 35 ± 12°) and (66 ± 10°,25 ± 11°) respectively.

Left-handed helices are understandably rare in proteins due to the inherent conformational preferences dictated by backbone stereochemistry. Less than one percent of residues are found in contiguous left-handed turns or helices of length three or greater. In the three-residue turns, the backbone angles progressively shift from being centered around the α_L _(*φ*, *ψ *≈ 60°,40°) to the 3_10-L _(*φ*, *ψ *≈ 70°,20°) (Figure 1). We do not detect a similar trend in four-residue structures although the number of examples is much smaller. Presumably this is due to the accommodation of an *i, i+3 *hydrogen bond in three-residue turns.

### Amino Acid Preferences in Left-handed Structures

Amino acid propensities at specific positions in the left-handed turns were computed as described in equations E1-E3 (Methods). The results, reported in Table 1, range from 0.0 - very unfavorable, to 1.0 - neither favorable nor unfavorable to 7.0 - very favorable. Due to the low counts and the very high frequency of Gly and Asn, which account for over a third of all residues in the data set, the 95% confidence intervals on many of the amino acids at specific positions are very large. The absolute values must therefore be interpreted very cautiously, and in cases where a favorable or unfavorable interaction is indicated from sequence statistics, the corresponding structures are also analyzed, or in some cases modelled using idealized structures.

**Table 1:** Mean amino acid propensities in three-residue left-handed turns and flanking positions.^a^

	**N"** **any conformation**	**N'** **not αL**	**Ncap** **αL**	**N1/C1** **αL**	**Ccap** **αL**	**C'** **not αL**	**C"** **any conformation**
ALA	0.52(0.26..1.89)	0.34(0.15..1.43)	0.86(0.42..2.69)	1.03(0.49..3.08)	0.86(0.42..2.69)	0.52(0.26..1.87)	1.55(0.66..4.24)

ARG	0.82(0.41..2.95)	0.54(0.24..2.24)	0.54(0.24..2.24)	0.27(0.02..1.51)	0.27(0.02..1.51)	1.34(0.66..4.19)	1.07(0.54..3.56)

ASN	0.66(0.29..2.73)	**2.59**(1.15..7.23)	5.49(1.86..13.47)	4.85(1.73..12.11)	2.91(1.25..7.95)	0.65(0.28..2.69)	0.32(0.03..1.82)

ASP	0.95(0.48..3.15)	1.17(0.57..3.66)	2.35(0.97..6.29)	0.94(0.47..3.11)	0.00(-0.38..0.48)	0.70(0.35..2.54)	1.64(0.75..4.73)

CYS	3.18(1.58..11.46)	1.04(0.09..5.88)	2.09(0.92..8.69)	1.04(0.09..5.88)	0.00(-1.68..2.13)	1.04(0.09..5.88)	2.09(0.92..8.69)

GLN	0.73(0.32..3.05)	0.36(0.03..2.03)	0.00(-0.58..0.74)	0.72(0.32..3.01)	1.08(0.54..3.91)	**3.61**(1.50..9.67)	0.36(0.03..2.03)

GLU	1.43(0.66..4.11)	0.81(0.40..2.67)	0.60(0.30..2.18)	0.20(0.02..1.13)	0.00(-0.32..0.41)	0.20(0.02..1.13)	0.40(0.18..1.68)

GLY	1.38(0.63..3.97)	1.17(0.55..3.48)	1.36(0.62..3.92)	2.91(1.04..7.28)	7.00(1.53..15.53)	1.55(0.69..4.35)	1.36(0.62..3.92)

HIS	0.61(0.05..3.41)	1.19(0.53..4.97)	1.19(0.53..4.97)	3.58(1.70..10.67)	0.00(-0.96..1.22)	1.19(0.53..4.97)	0.60(0.05..3.36)

ILE	0.49(0.22..2.03)	0.48(0.21..2.01)	0.24(0.02..1.36)	0.00(-0.39..0.49)	0.00(-0.39..0.49)	0.96(0.48..3.19)	0.48(0.21..2.01)

LEU	1.49(0.62..4.00)	0.88(0.42..2.64)	0.44(0.22..1.60)	0.59(0.30..1.95)	0.15(0.01..0.83)	0.29(0.13..1.23)	0.59(0.30..1.95)

LYS	0.00(-0.39..0.49)	0.24(0.02..1.34)	0.71(0.35..2.57)	0.48(0.21..1.98)	0.00(-0.38..0.48)	**1.90**(0.84..5.32)	0.95(0.48..3.15)

MET	2.45(1.22..8.83)	1.61(0.71..6.70)	0.80(0.07..4.53)	0.00(-1.29..1.64)	0.00(-1.29..1.64)	0.80(0.07..4.53)	0.80(0.07..4.53)

PHE	0.34(0.03..1.90)	1.33(0.67..4.42)	1.33(0.67..4.42)	1.00(0.50..3.61)	2.67(1.18..7.47)	0.33(0.03..1.88)	1.00(0.50..3.61)

PRO	0.31(0.03..1.72)	1.21(0.60..4.00)	0.00(-0.49..0.61)	0.00(-0.49..0.61)	0.00(-0.49..0.61)	0.30(0.03..1.70)	2.11(0.97..6.08)

SER	1.66(0.76..4.77)	0.94(0.47..3.10)	0.23(0.02..1.32)	1.40(0.67..4.18)	0.94(0.47..3.10)	1.40(0.67..4.18)	0.94(0.47..3.10)

THR	2.10(0.93..5.86)	**2.58**(1.07..6.92)	0.78(0.39..2.80)	0.00(-0.42..0.53)	0.00(-0.42..0.53)	**2.07**(0.92..5.79)	1.29(0.63..4.03)

TRP	0.00(-1.54..1.95)	0.00(-1.52..1.92)	3.78(1.89..12.50)	2.83(1.41..10.23)	0.94(0.08..5.32)	0.94(0.08..5.32)	0.94(0.08..5.32)

TYR	0.79(0.35..3.30)	1.57(0.78..5.18)	1.17(0.58..4.24)	0.78(0.34..3.26)	1.57(0.78..5.18)	0.78(0.34..3.26)	0.39(0.03..2.20)

VAL	0.99(0.48..3.07)	0.78(0.39..2.57)	0.19(0.02..1.09)	0.19(0.02..1.09)	0.00(-0.31..0.40)	0.58(0.29..2.11)	1.17(0.55..3.48)

^a ^Propensities greater than one indicate favorable interactions while values less than one indicate unfavorable interactions. Ranges in parentheses indicate 95% confidence intervals calculated using the Wilson interval score (ref [62]). Corresponding raw counts are in Additional Files 1: Table S2. Suggested N and C-capping motifs corroborated by structural data are highlighted in bold.

The highest propensities at the N1 - N3 positions belong to Gly and L-Asn. L-Asp is highly represented at the Ncap and N1 positions. These are also the three amino acids with the highest individual α_L _propensity in the database[20]. The preference of L-Asn (and L-Asp) for the α_L _has been suggested to result from favorable dipole-dipole interactions of sidechain and backbone carbonyls[38]. β-branched amino acids, L-Ile, L-Val and L-Thr are highly unfavorable. L-Pro is clearly not found in these structures due to the restriction of φ ≈ -60° by the cyclic sidechain.

Can propensities obtained from L-amino acids in α_L _turns provide insight into the thermodynamic effects of D-amino acids on α_R_-helix folding? To investigate this, database derived propensities were compared with experimental stabilities from host-guest studies (Table 2). Host-guest peptide systems have been used to quantify the helix stabilizing propensities of the various amino acids. This approach has been applied to both L-amino acids [39,40] and D-amino acids[41,42].

**Table 2:** Log propensities and thermodynamic scales of helix formation

	**P^a^**	**-log P**	**D scale^b^**	**L scale^c^**
GLY	3.81	-0.58	0.00	0.00
ASN	4.54	-0.66	-0.08	-0.01
CYS	1.68	-0.23	0.38	-0.22
TRP	3.07	-0.49	0.92	-0.45
HIS	1.94	-0.29	-0.15	0.03
ALA	1.01	0.00	1.05	-0.71
PHE	1.85	-0.27	1.15	-0.37
SER	0.99	0.00	0.47	-0.27
ASP	1.22	-0.09	0.42	-0.10
TYR	1.40	-0.15	1.36	-0.06
GLN	0.82	0.09	0.35	-0.33
MET	0.77	0.11	0.71	-0.42
LYS	0.54	0.27	0.82	-0.58
LEU	0.48	0.32	0.66	-0.52
THR	0.42	0.38	1.13	-0.09
ARG	0.52	0.28	0.69	-0.70
GLU	0.39	0.41	0.39	-0.21
VAL	0.25	0.60	1.16	-0.16
ILE	0.23	0.64	1.22	-0.17
PRO	0.19	0.72	1.44	

^a^Combined over Ncap, N1/C1 and Ccap of three-residue turns

^b^values from Krause, et al. [41]

^c^values from Betz, et al. [39]

**Figure 2:**
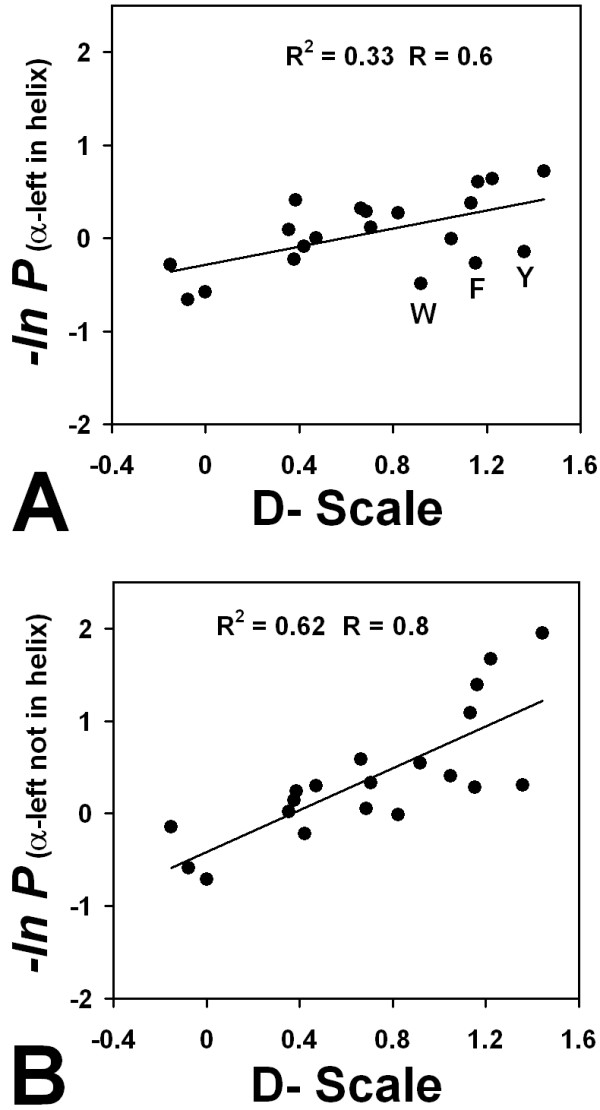
**Comparison of statistical propensities to thermodynamic scales for D-amino acids**. (A) log propensities for the twenty amino acids to occur in left-handed turns are plotted relative to the D-amino acid host-guest studies of Krause et. al. [43]. Line represents the best fit using linear regression. (B) log propensities were calculated for α_L _amino acids where preceding and following residues were not α_L_.

The influence of D-amino acid substitutions on the stability of an amphipathic, monomeric helix were studied by Krause and coworkers [43]. Comparing estimated statistical energies calculated as -*ln(P) *of combined propensities over the Ncap, N1/C1 and Ccap positions, to the Krause scale shows a reasonable correlation of approximately R = 0.58 (Figure 2A). The most distant outliers from the fit are the aromatic amino acids, Phe, Tyr and Trp. If these are omitted, the correlation improves: R = 0.85 (Table 3).

**Table 3:** Correlations of log-propensities and thermodynamic scales

	**All**	**I, T, V^a^**	**W, F, Y^a^**	**I, T, V, W, F, Y^a^**
-ln(P_αL-helix_) vs. D-scale	0.58	0.46	0.85	0.80
-ln(P_αL-not helix_) vs. D-scale	0.79	0.75	0.88	0.83
-ln(P_αR-helix_) vs. L-scale	0.73	0.73	0.74	0.75
-ln(P_αR-not helix_) vs. L-scale	0.42	0.41	0.44	0.43
L-scale vs D-scale	-0.41	-0.65	-0.43	-0.88

^a ^amino acids that were omitted from the fit

This strong correlation between database and experimental values is surprising, given the comparison of three-residue turns to the much longer eighteen-residue α-helix used in the host-guest studies. In an a-helix, an amino acid sidechain will often interact with i-3 and i-4 positions, either directly through van der Waals packing or hydrogen bonding, or indirectly through shielding of solvent interactions. It is possible that the host-guest scale is dominated by local stereochemical effects, rather than interactions with nearby residues that could have a cooperative effect on folding. To test this, a different set of database propensities were calculated using amino acids in an α_L _conformation where preceding and following amino acids were *not *α_L_. In this case, the correlation with the Krause scale also improves (R = 0.79 Figure 2B). This suggests that the experimental D-scale is describing the propensities of amino acids to assume backbone φ and ψ angles relating to an α_R _conformation, rather than reporting on steric interactions with i-3, i-4 positions in a helical context. Because monomeric helix folding-unfolding is not a two-state process [44,45], the amphipathic monomeric helix used may not reflect thermodynamic contributions in a larger protein where helix folding is coupled with assembly of other structural elements.

**Figure 3:**
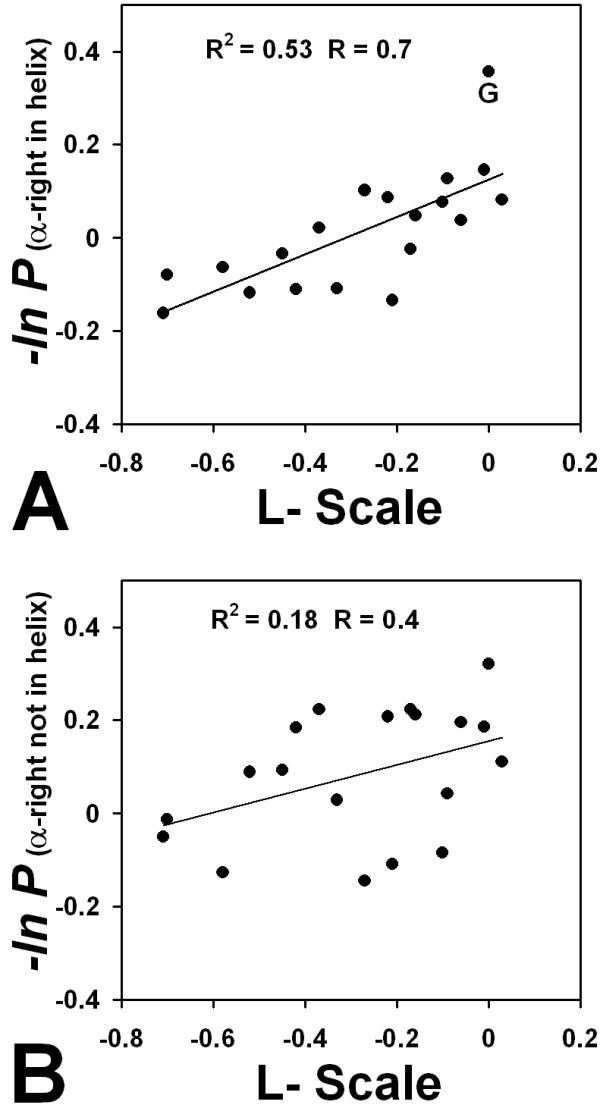
**Comparison of statistical propensities to thermodynamic scales for L-amino acids**. (A) log propensities for the twenty amino acids to occur in right-handed turns are plotted relative to thermodynamic scales from L-amino acid host-guest [39]. Line represents the best fit using linear regression. (B) log propensities were calculated for α_R _amino acids where preceding and following residues were not α_R_.

If the stereochemically inverse comparison is done, computing database α_R _propensities within a helix and in isolation, and correlating them with L-amino acid substitutions in a model two-state helical coiled-coil system [39], we now find that propensities in the helix (R = 0.73) correlate better with experimental values than those outside a helix (R = 0.42) (Figure 3). A similar result was observed for L-amino acids in right-handed helices by O'Neil et. al [40], who found a reasonable correlation (R = 0.75) between an experimental scale and propensities estimated from the PDB [46].

**Figure 4:**
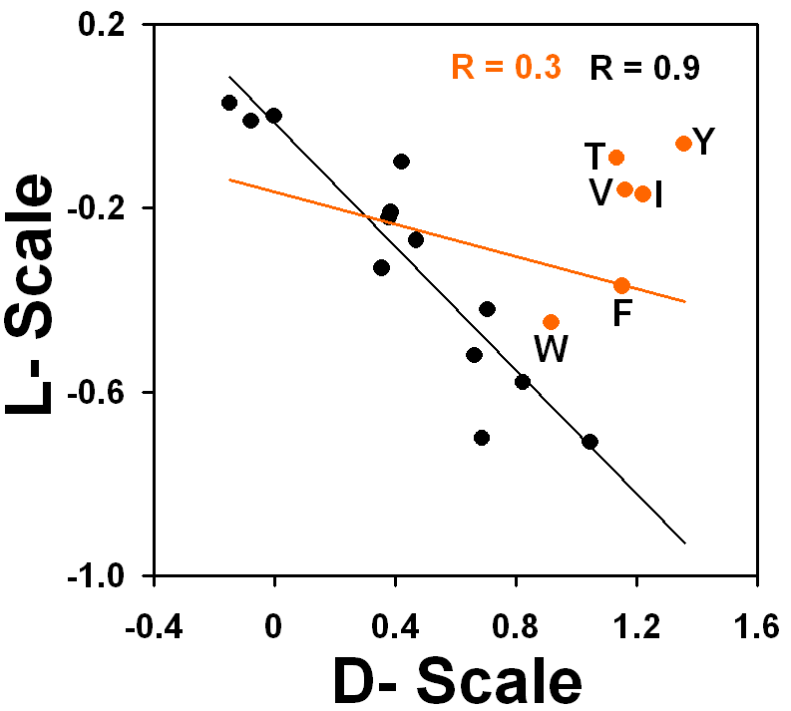
**Comparison of experimentally derived scales for L and D-amino acids**. Values from Krause et al. [43] and Betz et al. [39] were compared for all amino acids (black) and all except for aromatic and β-branched amino acids (orange).

In a direct comparison of the two experimental scales, the outliers are the β-branched amino acids (Ile, Val and Thr) and the aromatic amino acids (Phe, Tyr and Trp). When we remove these from the regression fit, the correlation improves from -0.41 to -0.88 (Figure 4). The six aromatic and β-branched amino acids are also the most highly ranked in several β-sheet propensity scales [47]. Thus, these particular residues are relatively unfavorable in a helix, regardless of its handedness because they favor the β_L _or β_R _region of conformational space, depending on their chirality. Less clear is the reason for the inverse correlation between α_L _and α_R _states of the remaining fourteen amino acids. It is possible that an L-amino acid that has both a low α_R _propensity and β_R _propensity will be more likely to occupy α_L_. Stabilization of one handedness is reflected in low occupancy of the other.

The amino acid with the lowest stability in α_R _helices is L-His [40]. Conversely D-His is one of the least destabilizing amino acids in α_R _helices [43]. L-His is observed with elevated frequency at the N1 position in this study. Assuming the neutral imidazole tautomer where Nδ^1 ^is deprotonated, histidine is the only other amino acid beside Asn and Asp that presents a lone pair separated by three bonds from the Cα carbon on the backbone. If the dipole-dipole interaction between backbone and sidechain carbonyls suggested for L-Asp and L-Asn [38] can stabilize the α_L _conformation, one may speculate that a similar mechanism may be at work in the case of the imidazole Nδ^1 ^lone pair and its dipolar interaction with the backbone carbonyl.

### Backbone Conformations for Positions Flanking a Left-handed Turn

**Figure 5:**
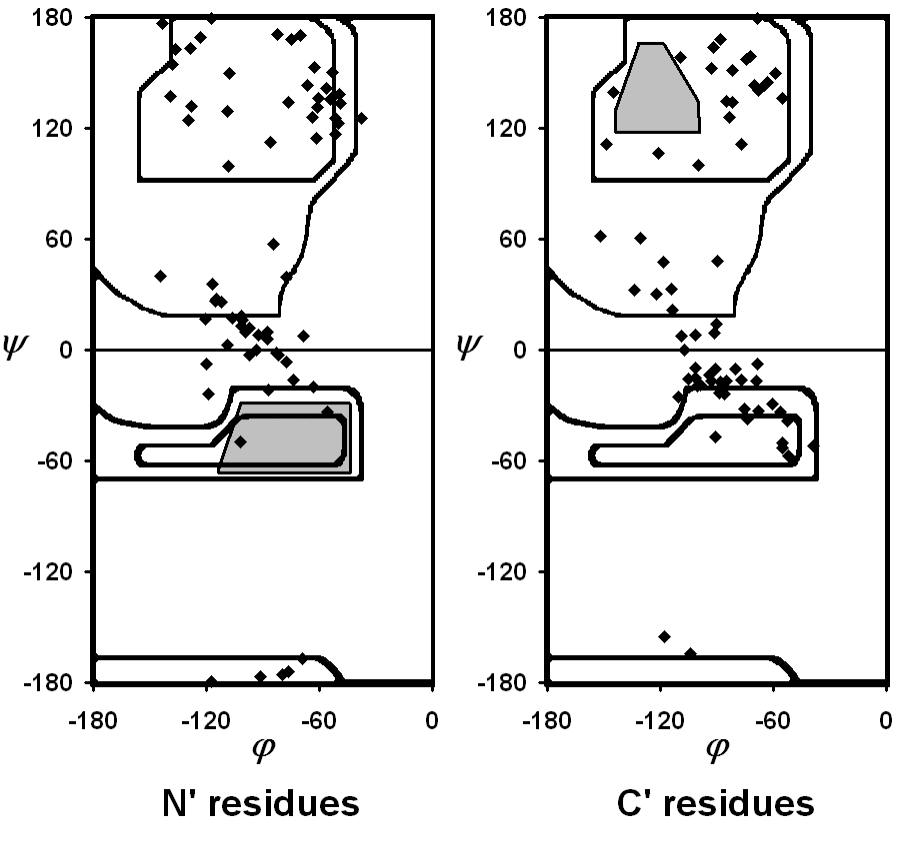
**Backbone conformational preferences for positions flanking an α_L _turn**. Backbone conformations for the N' and C' positions of the three-residue left-handed turns. Excluded regions of β in the C' and α_R _in the N' are shaded.

As defined in this study, the N' and C' positions are the amino acids directly preceding and following the left-handed turn. Most of these fall in the α_R _and β_R_/polyproline II (PP2) regions of Ramachandran space (Figure 5). Certain regions are sparsely occupied. These empty regions differ in the context of the N and C-termini. At N', residues are in the γ_R _(*ψ *> 0°) rather than the α_R _region (*φ *≈ -65°, *ψ *≈ -40°). At the C', residues are rarely found in standard β_R _conformations and instead primarily occupy the PP2 region.

**Figure 6:**
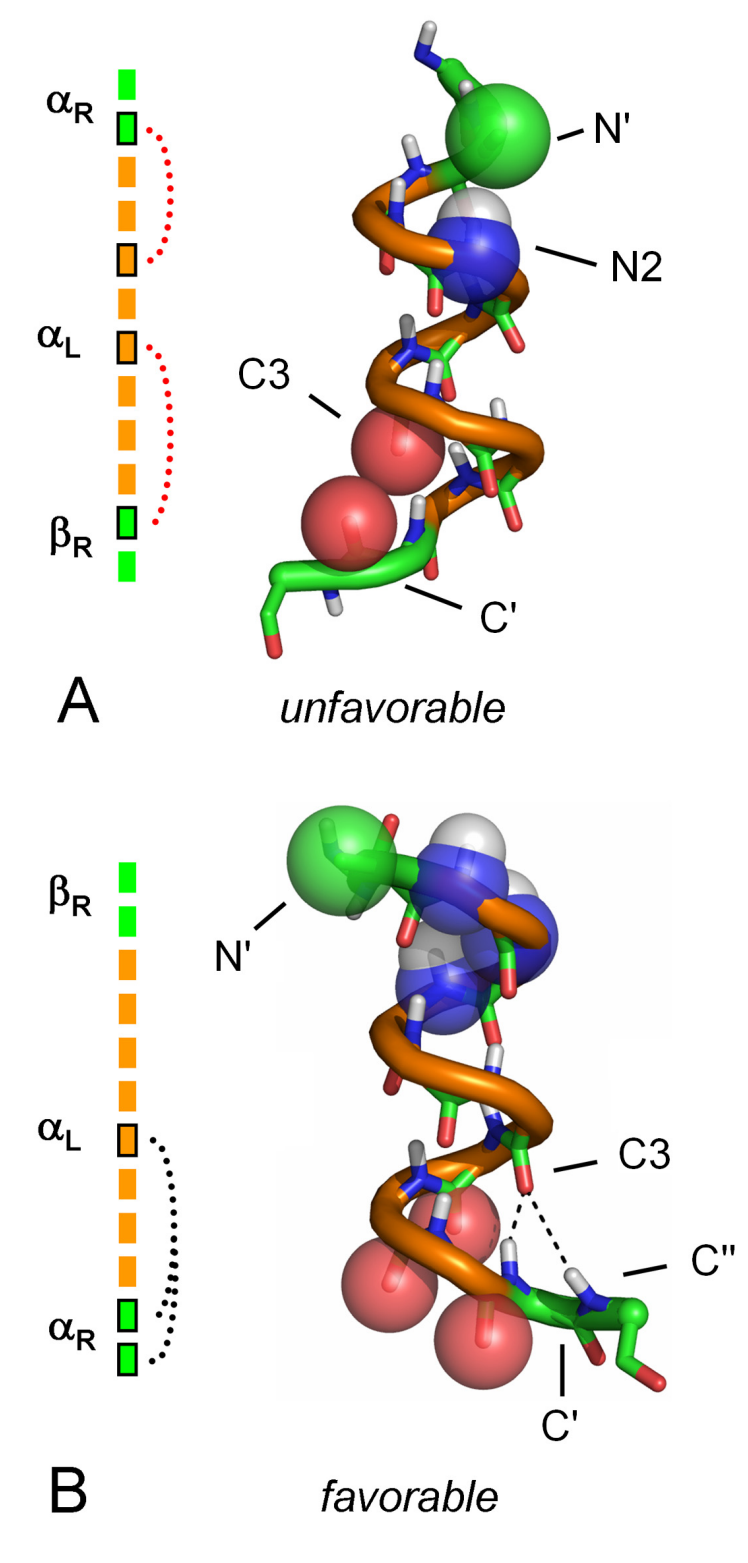
**Modeling favorable and unfavorable helix flanking conformations**. Stereochemical constraints on flanking positions of a model α_L _helix (φ = 65°, ψ = 42°). (A) Unfavorable flanking conformations. Placing an Ncap residue in the α-R conformation occludes solvation of the N2 amide by the N' sidechain. A C' residue in the β_R _conformation causes a steric clash between the C' and C3 carbonyls. (B) Favorable flanking conformations. An N' in the β_R _conformation removes any desolvation of the Ncap-N2 amides (shown as spheres). An α_R _C' replaces the carbonyl clash to C3 with a bivalent hydrogen bond from the C' and C" amides. Carbons of residues in the α_L _conformation are colored orange.

These unoccupied areas can be used to develop rules of conformational exclusion surrounding an α_L _helix. Similar rules have been developed in studies by Fitzkee and Rose, who found that an α_R _helix is not followed by certain regions of β [48,49]. In the right-handed helix, steric clashes between the C' carbonyl and that of a neighboring carbonyl from the C2 position of the helix prevent α_R _being followed directly by a β-strand[48]. In a left-handed helix, modeling suggests a similar constraint is enforced by a repulsive interaction between the C' and C3 carbonyl groups (Figure 6). This prevents the β_R_-strand conformation from following an α_L _helix. Placing the C' amino acid in α_R _or poly-proline II (PPII) relieves this steric clash. For C-capping residues in the α_R _conformation, a Schellman-like capping interaction is possible, allowing for hydrogen bonds from the C' and C" backbone amides to the C3 carbonyl.

At N', the α_R _conformation is disallowed. When a helix is modeled with an α_R _residue followed by an α_L _helix, no strong steric clash is observed (Figure 6). However, the Cβ sidechain methyl of the N' residue prevents solvation of the N2 backbone amide. Desolvation of polar groups are energetically unfavorable when no intrinsic hydrogen bond within the protein replaces the interaction[50]. This desolvation penalty can be partially relieved by placing the N' in either the β_R_, PPII or γ_R _conformation. Thus, two conformational rules unique to flanking positions of left-handed helices emerge: α_R_-(α_L_)_n _and (α_L_)_n_-β_R _are disfavored where n ≥ 3. Similar rules would apply to the structural enantiomer where D-amino acids precede or follow an α_R _helix: α_L_-(α_R_)_n _and (α_R_)_n_-β_L _would be disfavored for n ≥ 3.

### Sidechain-Backbone Interactions at the N-terminus

If the N' residue is in the β_R _conformation as pictured in Figure 6B, unfavorable desolvation of the N2 amide is avoided, but the N' sidechain projects away from the top of the helix, preventing any specific polar capping interactions with the N-terminal amides. Such capping interactions are prevalent in α_R _helices which often feature L-Thr, L-Asn or L-Asp at the N-terminus making sidechain oxygen acceptor hydrogen bonds to exposed backbone amides[27,28]. To accommodate this, the capping residue is usually in the β conformation. A similar propensity for small polar amino acids at the N' is observed in our database of left-handed turns. However, for these to facilitate sidechain-backbone capping hydrogen bonds while avoiding desolvation of N2, the residue must be in the γ_R _(*ψ *> 0°) conformation. Although both α_R _and α_L _N-terminal capping interactions involve small polar amino acids, the interactions presented here are structurally distinct from those previously identified

**Figure 7:**
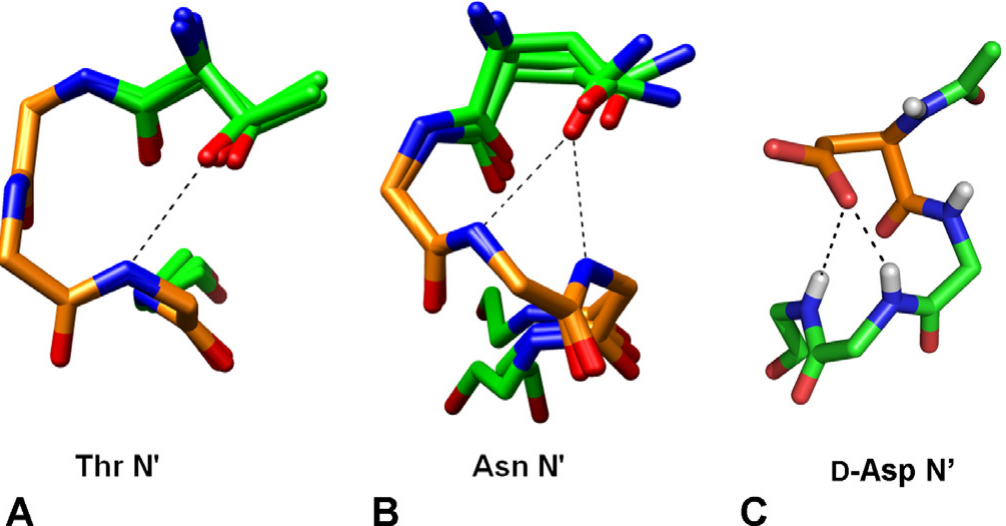
**N' capping interactions**. (A) N' Thr and (B) Asn contribute sidechain-backbone hydrogen bonds. Carbons in α_L _turn are orange, others are in green. Sidechain atoms are only shown for the capping residues. Thr caps shown: 1ZY7_A 339-343, 1OVM_A 292-296 and 1GSA 188-192. Asn caps shown: 1AA7_A 85-89, 1P4C_A 254-258 and 1KQF_A 521-525. (C) The D-Asp capping interaction for an α_R _helix from a designed peptide (CSD ID - GORVIP) [51]. D-Asp carbons are colored orange.

L-Asn and L-Thr show an elevated propensity to occur at the N' position. N' residues in the γ_R _conformation are enriched for small, polar amino acids. L-Asn and L-Thr in the γ_R _conformation adopt rotamers that allow hydrogen bonding between the sidechain oxygen and the N1 and N2 amides (Figure 7A, B). The χ_2 _rotamer angle places the sidechain oxygen rather than nitrogen over the terminus, consistent with L-Asn functioning as a hydrogen bond acceptor. In this configuration, the sidechain oxygen also forms a hydrogen bond with its own backbone amide, contributing further to the stability of this motif.

In a designed turn-helix peptide, a D-Asp was utilized to contribute similar interactions at the N-terminus of an α_R _helix (Figure 7C) [51]. These N-terminal interactions are a subset of a larger class of motifs in proteins and peptides described by Milner-White and colleagues as peptide 'nests' [30,52]. These nests often serve as anion binding sites, complexing both sidechains and prosthetic groups such as phosphates and iron-sulfur clusters [53,54].

### Sidechain-Backbone Interactions at the C-terminus

**Figure 8:**
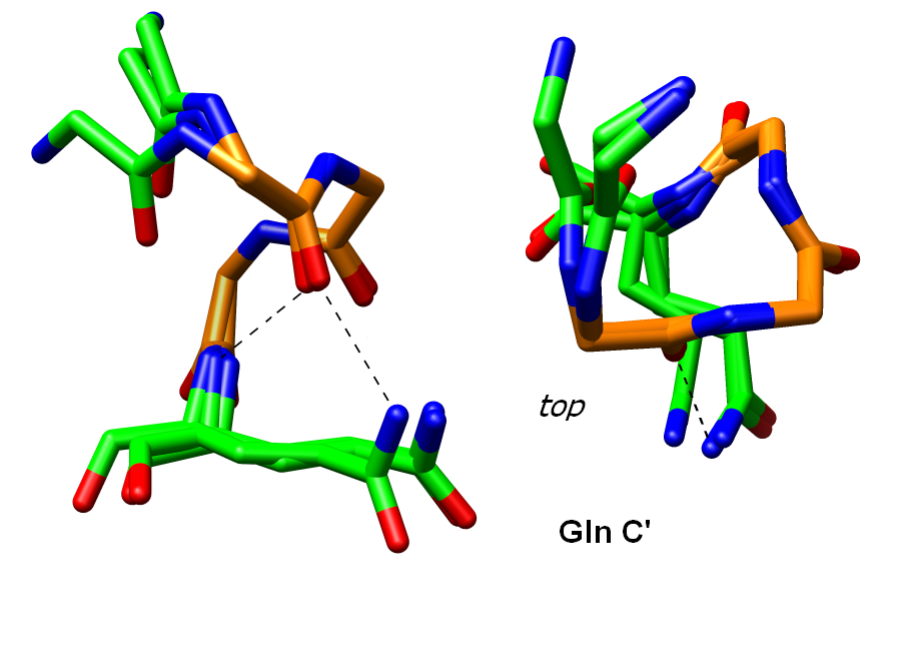
**C' Gln capping of α_L _turns**. Several examples are found in the PDB of C-capping interactions involving C' Gln in the α_R _conformation. Carbons for the α_L_-turn are in orange, others are in green. Sidechain atoms are shown for the capping residues only. Structures shown are: 2J6L_A 297-301, 1A4S_A 291-295 and 1EZ0_A 283-287.

**Figure 9:**
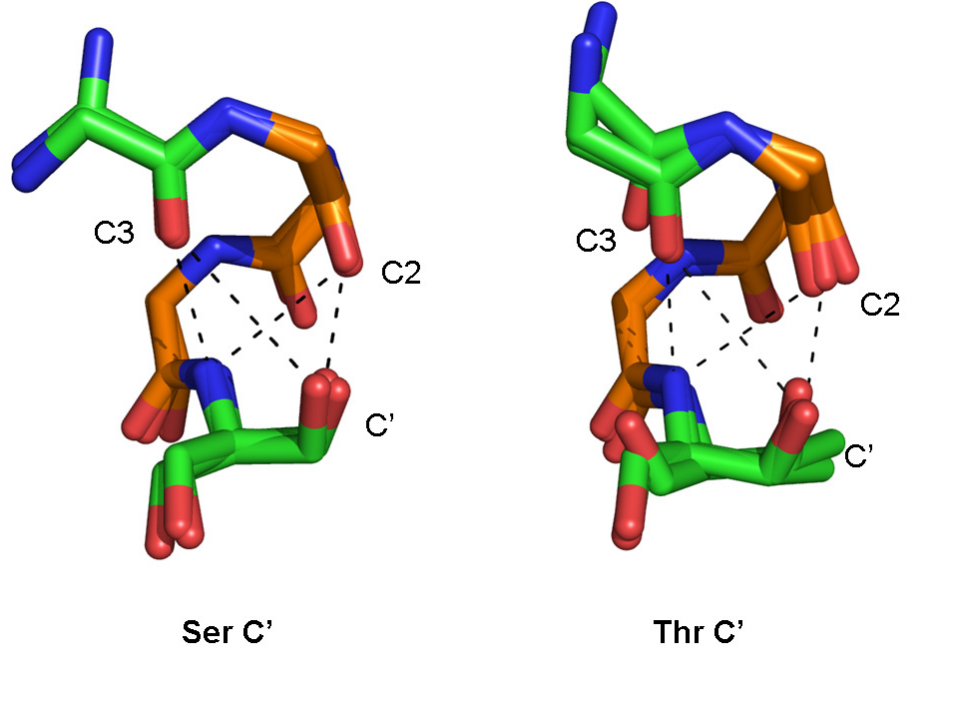
**C' Ser and Thr capping of α_L _turns**. Ser and Thr mediated C' hydrogen bonds. α_L_-turn carbons are colored orange and the flanking residues are green. Sidechain atoms are shown for the capping residues only. Ser structures shown: 2FFU_A 525-529, 1ZY7_A 340-344 and 1MD6_A 61-65. Thr structures shown: 1UYL_A 107-111,1HYO_A 368-372 and 1GSA_A 190-194.

The majority of C' amino acids in our survey of three residue left-handed turns are found in the α_R_/γ_R _conformation. This facilitates formation of Schellman-like interaction between the C' amide and the carbonyl of the C2 position. In α_R _helices, the Schellman capping motif often involves glycine which readily adopts the α_L _conformation[55]. In α_L _helices, an α_R _cap is facilitated by the chirality of L-amino acids, avoiding the entropic cost associated with fixing the conformation of glycine. We looked for additional stabilization of these Schellman-like caps through sidechain-backbone interactions. The highest propensity at the C' is L-Gln which occurs 3.6-fold more often than random expectation. An analysis of structures with a C-terminal L-Gln shows a bivalent hydrogen bond to the C2 carbonyl from both the backbone and sidechain amide (Figure 8). This effect is very specific for L-Gln and a similar propensity is not observed for L-Asn. L-Thr and L-Ser also make capping interactions at the C-terminus of left-handed helices. A similar bivalent hydrogen bond is accepted by the C2 and C3 carbonyls from the C' sidechain hydroxyl and backbone amide (Figure 9). L-Lys is also elevated at the C' position, suggesting stabilization of a helix macrodipole, although L-Arg does not have a high propensity at this position.

It is interesting to compare our observations with studies on the energetics of C-terminus helix capping through chemical synthesis of proteins with D-amino acids. Bang and coworkers replaced Gly 35 of ubiquitin, which sits in the α_L _conformation at the end of an α_R _helix, with D-Ala, D-Gln, D-Val and D-Thr[34]. D-Ala and D-Gln have comparable stabilities and are both very close to the stability of the wild type Gly 35 protein. The β-branched amino acids are less stable by nearly 1 kcal/mol. D-Val is less stable than D-Thr by approximately 0.5 kcals/mol. Although the study states that these energy differences relative to glycine correlate with changes in solvation of the carboxy terminus, it is possible that specific interactions such as the ones we observe are also contributing to capping energetics. This would explain the increased stability of D-Thr over D-Val, which has the facility to form Ccap hydrogen bonds in the α_L _conformation to an α_R _helix. The similarity in energetics of D-Gln and D-Ala show that in ubiquitin, D-Gln sidechain capping interactions are not playing a significant role in protein stabilization. In high resolution structures of the D-Gln 35 mutant, the sidechain does not make the same capping interaction we observe, but rather is involved in quaternary contacts with other ubiquitins in the asymmetric unit[1,34]. With three rotameric degrees of freedom, Gln has to pay a higher entropic cost to form the specific hydrogen bond to the C2 carbonyl. This may cancel the energy gained by forming a capping hydrogen bond. We have recently shown that Gly to D-Gln mutations can significantly increase the stability relative to the D-Ala substitution of the Trp-Cage. (manuscript in preparation).

### Stabilization Through Tertiary Interactions

**Figure 10:**
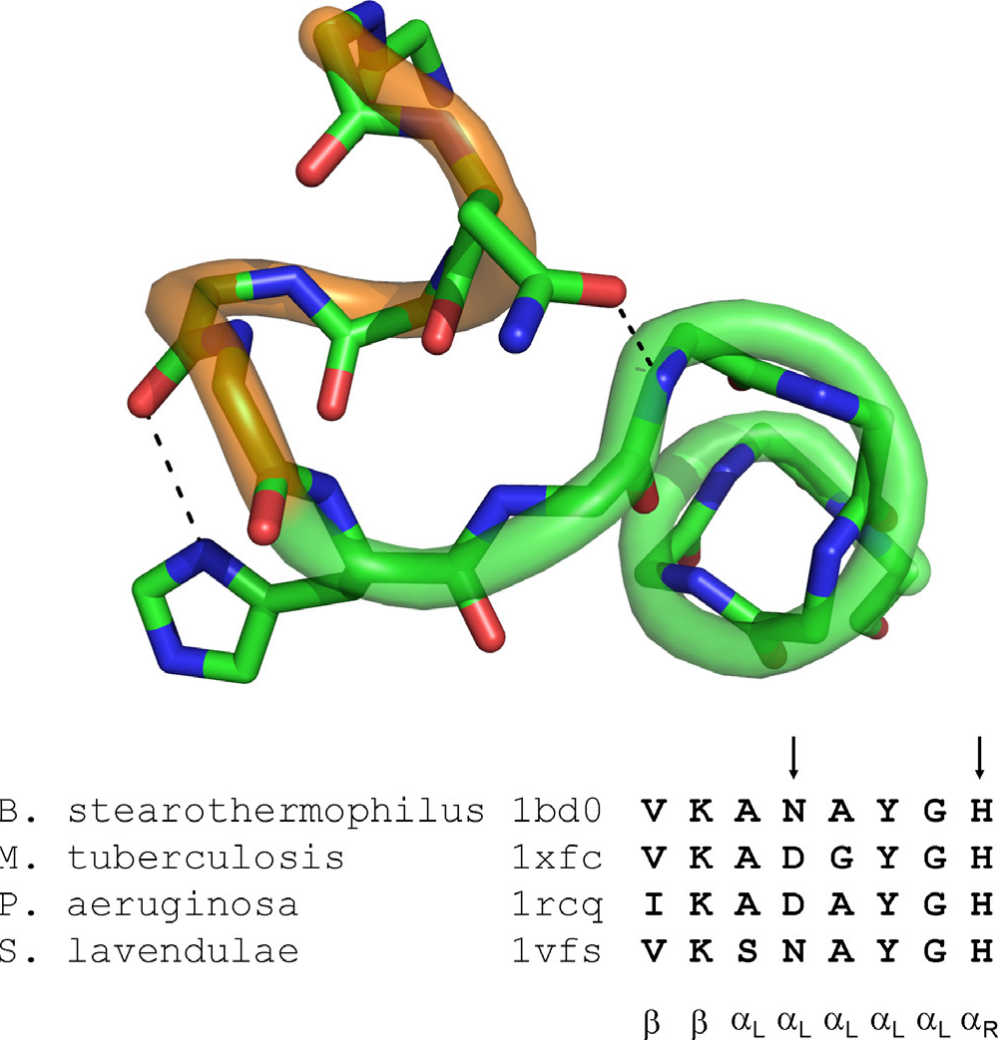
**An α_L_-helix-turn-α_R_-helix in alanine racemase**. Conserved interactions across multiple bacterial species include a histidine α_L_-helix C' and a tertiary Asn/Asp hydrogen bond to the N-terminus of the α_R_-helix.

Two examples of five-residue left-handed helices are in our database. Alanine racemase is an enzyme which catalyzes the conversion of L-Ala to D-Ala and plays an important role in bacterial cell wall synthesis. Residues 40-44 in alanine racemase from *B. stearothermophilus *(PDB 1BD0) form a contiguous α_L _helix [56]. This feature was originally noticed by Kleywegt using a spatial motif search [57]. Strong sequence conservation maintains this structural motif across several other bacterial species (Figure 10). L-Lys 39 and L-Tyr 43 are part of the enzyme active site and are functionally conserved positions[58]. L-His 45 serves as a Schellman-like C' in the α_R _conformation with an additional hydrogen bond between the imidazole Nδ^1 ^and the carbonyl of the C1 position. An additional stabilizing hydrogen bond is provided by L-Asp/L-Asn 41 to the N-terminus of an adjacent right-handed helix. This interaction serves both to stabilize the α_L _helix and specify the helix-bend-helix conformation. Bent motifs with adjacent helical structures of opposite handedness and chirality were found in previous simulations of heterochiral secondary structures in poly-alanine[15] and in the molecular structure of tolaasin[12]. A specific hydrogen bond such as the one provided by residue 41 could be used in the design of *de novo *heterochiral helical bends.

**Figure 11:**
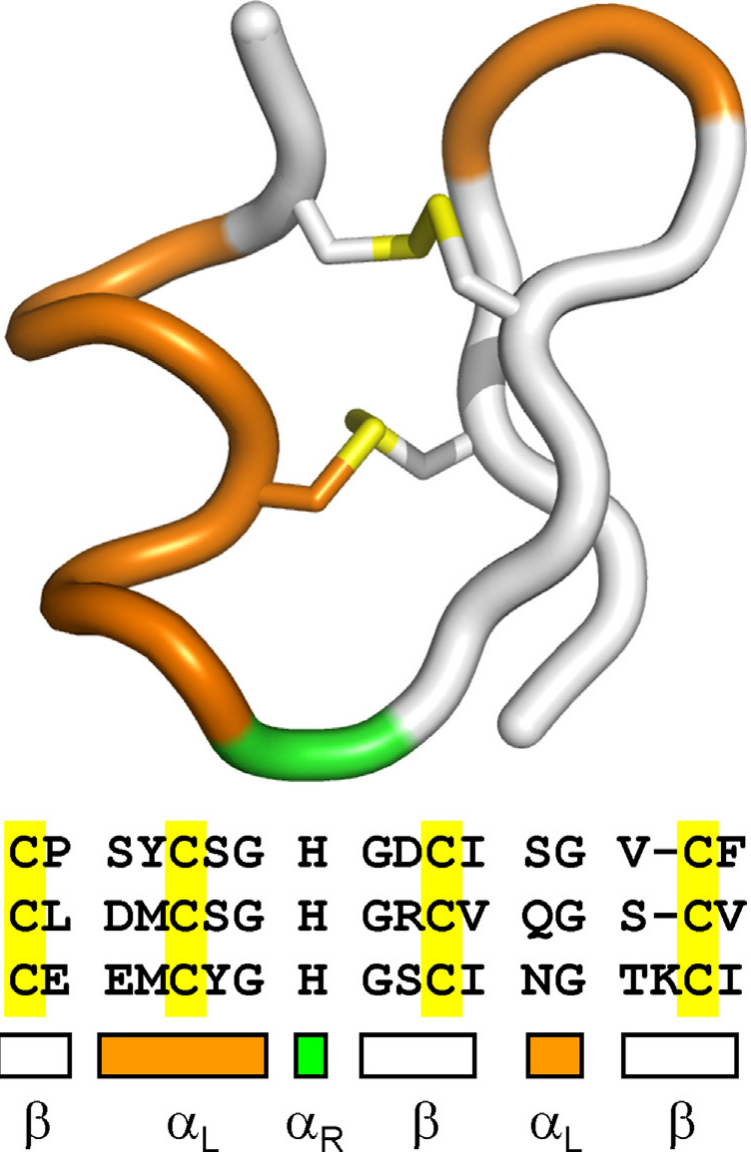
**A repeated 3_10-L_-helix - b-hairpin in reelin**. A pair of disulfides with the hairpin maintains the five-residue left-handed helix.

The other five residue motif achieves stability through disulfides. Three of the repeat domains in reelin, a protein involved in neurological development, have been shown to contain a five residue 3_10-L _helix[59]. L-Cys at the N" and N2 position participate in disulfides with an adjacent β-hairpin (Figure 11). Although L-His is found at the capping position in all three reelin repeat domain structures, it does not participate in sidechain-backbone capping interactions as was observed in alanine racemase. This structure provides a useful exemplar upon which to design novel α_L_-helix-β-hairpin folds.

## Availability and requirements

The PERL script used to identify α_L _regions is included as Additional Files 2: *findalphaleft.pl*

## Conclusion

To make the rational engineering and design of heterochiral proteins tractable, the role of amino acid stereochemistry in stability and structure needs to be better understood. This study presents potential rules based on insights gained from the analysis of natural proteins. Using left-handed turns and helices in the database of existing protein structures as a case study, we have found several candidates for motifs that could be applied to the thermostabilization of proteins by synthetic amino acids. As synthetic methods for building proteins continue to improve, the ability to construct larger molecules with mixtures of natural and synthetic amino acids becomes increasingly practical. Natural proteins can provide important insight into how designed proteins can take advantage of the increased chemical diversity made possible by synthetic methods.

## Methods

### Compiling a non-redundant set of PDB files

A list of non-redundant protein chains was assembled using PISCES [60,61]. Structures obtained through X-ray crystallography with a resolution greater than 2.5 Å and sequence homology less than 25% were included. The final database consisted of 3517 unique chains.

### Searching for α_L _helices

PERL scripts were constructed to search each file on the non redundant list for presence of α_L _helices of three residues or longer (see Additional Files 2). φ and ψ values were computed based on deposited backbone coordinates of the N, C, Cα and O atoms (see scripts for details). *φ *values between 35.0° and 95.0° and *ψ *values between 10.0° and 70.0° were classified as α_L_. Allowable ranges were settled on after starting with more generous ranges and narrowing the window until all structures showed i, i+3 and or i, i+4 hydrogen bonding (determined geometrically by checking the backbone N to O distance was less than or equal to 3.5Å). Initially, the search returned eighty-five α_L_-helices and turns of which seventy-three were three residues long, ten were four residues long and two were five residues long.

In order to assess local structure quality, backbone B-factors were examined for the three-residue α-turns in our data set. Three turns in our data set with B-factors greater than one standard deviation from the mean were flagged for manual examination. WinCoot was used to visualize electron density maps based on structure factors deposited at the EDS. One structure for which there was poor electron density in the turn region was removed from the data set (see Additional Files 1: Figure S1).

### Calculating amino acid propensities

Sequences of the three residue left-handed turns were analyzed to determine amino acid propensities at each position in the turn. The occurrence of an amino acid at each position was divided by occurrence in the PDB dataset to obtain normalized values.

For three-residue left-handed turns, propensities of the twenty amino acids were calculated for the N', Ncap, N1/C1, Ccap and C' positions (Table 1). Propensities for each amino acid type *aa*_i _at position *pos*_j _were normalized to total occurrence in the database:

(1)

(2)

(3)

The propensity for a particular amino acid to occur in the Ncap, N1 or Ccap position was compared to the overall frequency of that amino acid type in the α_L _conformation in any context. Overall frequencies were calculated using the same data set of proteins from which the left handed turns were selected.

The contribution of sampling error to the mean and 95% confidence intervals was estimated using a Wilson score interval for the counts in helices [62]. Corrected values are reported in Table 1.

## Authors' contributions

SA carried out the research and analyzed the data. SA and VN wrote the paper.

## Supplementary Material

Additional file 1 **Supplementary Figures and Tables**. This file contains additional tables and figures. Table S1: Left-handed turn-containing structures, Table S2: Residue counts in three-residue turns and Figure S1: Electron Density Maps of Relative High B-factor Turns. Click here for file

Additional file 2 **PERL script code**. *findalphaleft.pl *is the PERL script used to identify α_L_-helices and turns in the PDB. Click here for file
