# Supplementary material for: Mirrors in the PDB: left-handed α-turns guide design with D-amino acids
Source: BMC Struct Biol. 2009 Sep 22;9:61. doi: 10.1186/1472-6807-9-61 (PMC2759939; doi:10.1186/1472-6807-9-61)
Supplement: Additional file 1 — Supplementary Figures and Tables. This file contains additional tables and figures. Table S1: Left-handed turn-containing structures, Table S2: Residue counts in three-residue turns and Figure S1: Electron Density Maps of Relative High B-factor Turns. [file 1472-6807-9-61-S1.DOC]

# Supplementary Information: Mirrors in the PDB: left-handed α-turns guide design with D-amino acids

### Srinivas Annavarapu, Vikas Nanda

## Table S1 – Turn containing structures

A list of all non‑redundant PDB files containing three or more continuous residues in α_L_ conformation are listed. The sequence listed includes two flanking residues at N and C‑terminus with residues in α_L_ conformation marked by a box.

*Three residue turns*

| **PDB ID** | **Chain** | **Start** | **End** | **Sequence** |
| --- | --- | --- | --- | --- |
| 2bz1 | A | 93 | 95 | QEGRNIG |
| 2a2l | A | 98 | 100 | LTNQQRI |
| 1sqj | A | 372 | 374 | LDFNNGP |
| 2eut | A | 37 | 39 | EYDNYIG |
| 2eab | A | 414 | 416 | TPWGSDF |
| 2hc1 | A | 1698 | 1700 | QADSNYL |
| 1snr | A | 105 | 107 | TGALGGG |
| 1yuk | B | 437 | 439 | SLCHGKG |
| 1ez0 | A | 284 | 286 | TMGCGQF |
| 2f9i | A | 124 | 126 | YRNFGMA |
| 1uar | A | 88 | 90 | GDKNNWW |
| 2yqc | A | 185 | 187 | ENNYFGL |
| 1tht | A | 49 | 51 | RMDHFAG |
| 1ds1 | A | 115 | 117 | ELRSGTV |
| 2nzl | A | 260 | 262 | SNHGARQ |
| 1d2o | A | 548 | 550 | DDKDNQD |
| 2ppv | A | 84 | 86 | NQVDGHS |
| 2ga1 | A | 40 | 42 | GVCGGQA |
| 2fkc | A | 226 | 228 | GGDNGRE |
| 1n62 | B | 389 | 391 | RCSFRVT |
| 2boq | A | 30 | 32 | LFDGAQC |
| 1m22 | A | 170 | 172 | SEWANFR |
| 1xmt | A | 32 | 34 | MRNNGKV |
| 1vef | A | 52 | 54 | CVGGYGV |
| 2ivf | C | 143 | 145 | MKDKAGS |
| 1thf | D | 143 | 145 | FTYSGKK |
| 1jix | A | 191 | 193 | SFRSGQR |
| 1aa7 | A | 86 | 88 | LNGNGDP |
| 1uyl | A | 106 | 108 | INNLGTI |
| 1m1n | A | 444 | 446 | HSWDYSG |
| 1ceo |  | 175 | 177 | GNNYNSP |
| 1n7v | A | 123 | 125 | ITNGGNP |
| 2phn | A | 105 | 107 | CVNAGID |
| 1v58 | A | 208 | 210 | MSKENTL |
| 1hyo | A | 367 | 369 | LSWKGTK |
| 1bqc | A | 166 | 168 | APNWGQD |
| 2cjl | A | 70 | 72 | ETGGLVY |
| 1zpd | A | 293 | 295 | STTGWTD |
| 2o62 | A | 42 | 44 | GLNNNQT |
| 1lf6 | A | 290 | 292 | NNFNGKA |
| 1o97 | D | 135 | 137 | GGYNQKV |
| 1g3k | A | 38 | 40 | RLYNGKV |
| 2i8g | A | 4 | 6 | VHDSALP |
| 2eb4 | A | 165 | 167 | SDNAANA |
| 1olz | A | 108 | 110 | GTNAFQP |
| 2gc7 | A | 53 | 55 | DPAHFAA |
| 2e7z | A | 690 | 692 | ETGFSGA |
| 7a3h | A | 177 | 179 | TGTWSQD |
| 2fju | B | 645 | 647 | EFNGQSG |
| 1on3 | A | 83 | 85 | VPADGVV |
| 1on3 | A | 108 | 110 | TVMGGSA |
| 2ii0 | A | 910 | 912 | SEDHYKK |
| 2j6g | A | 45 | 47 | LTNGGTK |
| 2ofk | A | 61 | 63 | CFHQFDP |
| 1ah7 |  | 62 | 64 | YYDNSTF |
| 2j6l | A | 298 | 300 | VGTAGQR |
| 1wpn | A | 151 | 153 | DSLLFKS |
| 1gsa |  | 189 | 191 | LTEHGTR |
| 1zy7 | A | 340 | 342 | LTDNFSS |
| 1u7l | A | 39 | 41 | TLIGGRA |
| 2oaj | A | 418 | 420 | PYFAGCH |
| 1kqf | A | 522 | 524 | ENNWGYD |
| 2bdr | A | 32 | 34 | FINNGST |
| 1v4s | A | 316 | 318 | LLFHGEA |
| 2biw | A | 170 | 172 | DDLGGIL |
| 1x3l | A | 206 | 208 | AIASGPT |
| 1ak0 |  | 131 | 133 | AYAVGGN |
| 1wm1 | A | 239 | 241 | THLGFLE |
| 1uc2 | A | 171 | 173 | LEEGGRM |
| 2f2b | A | 131 | 133 | VGGLGAT |
| 1z6o | A | 192 | 194 | TANNGHD |
| 2bz6 | L | 94 | 96 | VNENGGC |

Four residue helices

| **PDB ID** | **Chain** | **Start** | **End** | **Sequence** |
| --- | --- | --- | --- | --- |
| 1vpr | A | 973 | 976 | LCFNNFQD |
| 1v84 | A | 301 | 304 | PKAANCTK |
| 2oo4 | A | 1449 | 1452 | ACQWDGGD |
| 2oo4 | A | 1528 | 1531 | ECGWDGLD |
| 2e26 | A | 2484 | 2487 | CLDMCSGH |
| 2gzq | A | 163 | 166 | RVKFNNCQ |
| 2qah | A | 180 | 183 | IDHMGRPD |
| 1koe |  | 266 | 269 | SYCETWRT |
| 2qe8 | A | 85 | 88 | DNGNQSKS |
| 1rcq | A | 34 | 37 | IKADAYGH |

Five residue helices

| **PDB ID** | **Chain** | **Start** | **End** | **Sequence** |
| --- | --- | --- | --- | --- |
| 2oo4 | A | 1490 | 1494 | ECLFDNFEC |
| 2e26 | A | 2135 | 2139 | CEEMCYGHG |

***Table S2 – Residue Counts in Three-Residue Turns***

|  | **N’’’** | **N’’** | **N’** | **Ncap** | **N1/C1** | **Ccap** | **C’** | **C’’** | **C’’’** |
| --- | --- | --- | --- | --- | --- | --- | --- | --- | --- |
| **ALA** | 4 | 3 | 2 | 5 | 6 | 5 | 3 | 9 | 9 |
| **ARG** | 8 | 3 | 2 | 2 | 1 | 1 | 5 | 4 | 1 |
| **ASN** | 4 | 2 | 8 | 17 | 15 | 9 | 2 | 1 | 6 |
| **ASP** | 5 | 4 | 5 | 10 | 4 | 0 | 3 | 7 | 2 |
| **CYS** | 2 | 3 | 1 | 2 | 1 | 0 | 1 | 2 | 2 |
| **GLN** | 1 | 2 | 1 | 0 | 2 | 3 | 10 | 1 | 2 |
| **GLU** | 6 | 7 | 4 | 3 | 1 | 0 | 1 | 2 | 4 |
| **GLY** | 3 | 7 | 6 | 7 | 15 | 36 | 8 | 7 | 6 |
| **HIS** | 1 | 1 | 2 | 2 | 6 | 0 | 2 | 1 | 2 |
| **ILE** | 4 | 2 | 2 | 1 | 0 | 0 | 4 | 2 | 3 |
| **LEU** | 6 | 10 | 6 | 3 | 4 | 1 | 2 | 4 | 8 |
| **LYS** | 3 | 0 | 1 | 3 | 2 | 0 | 8 | 4 | 1 |
| **MET** | 2 | 3 | 2 | 1 | 0 | 0 | 1 | 1 | 2 |
| **PHE** | 4 | 1 | 4 | 4 | 3 | 8 | 1 | 3 | 3 |
| **PRO** | 3 | 1 | 4 | 0 | 0 | 0 | 1 | 7 | 3 |
| **SER** | 3 | 7 | 4 | 1 | 6 | 4 | 6 | 4 | 2 |
| **THR** | 2 | 8 | 10 | 3 | 0 | 0 | 8 | 5 | 3 |
| **TRP** | 2 | 0 | 0 | 4 | 3 | 1 | 1 | 1 | 2 |
| **TYR** | 4 | 2 | 4 | 3 | 2 | 4 | 2 | 1 | 5 |
| **VAL** | 4 | 5 | 4 | 1 | 1 | 0 | 3 | 6 | 5 |
| **n** | 71 | 71 | 72 | 72 | 72 | 72 | 72 | 72 | 71 |

***Figure S1: Electron Density Maps of Relative High B-factor Turns***


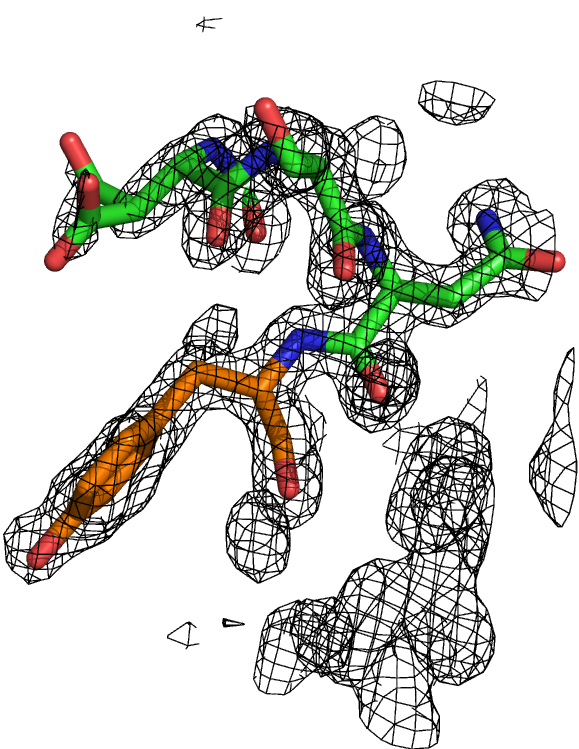
**(a)** 2HC1 – residues 1698-1700 fell within the 2F_0_-DF_c_ map. This turn was included in the analysis.


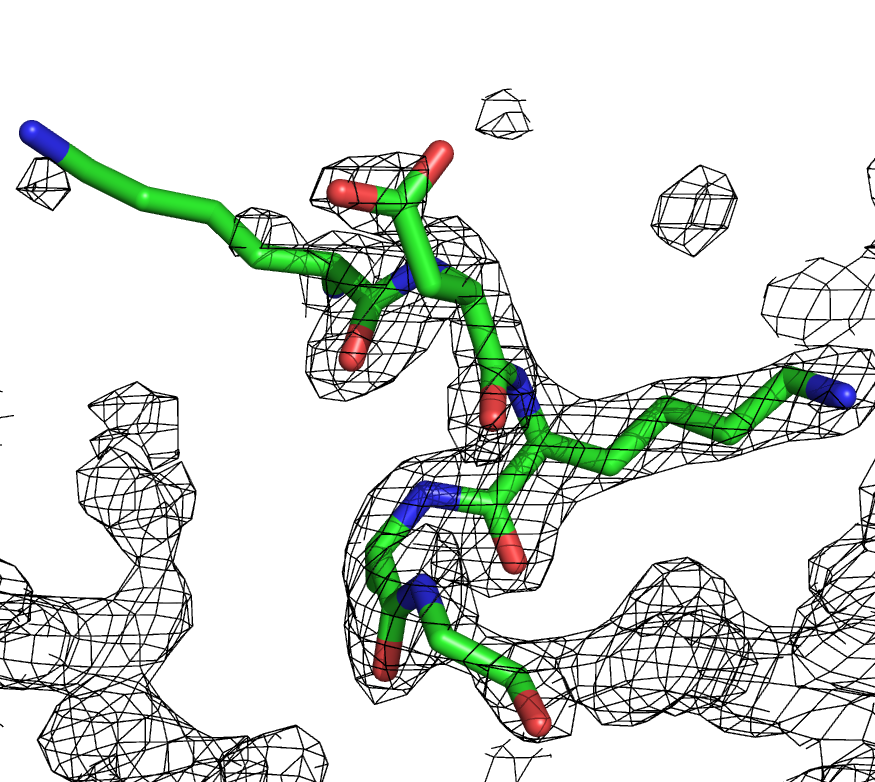


**(b)** 2IVF – residues 143-145 fell within the 2F_0_-DF_c_ map. This turn was included in the analysis.


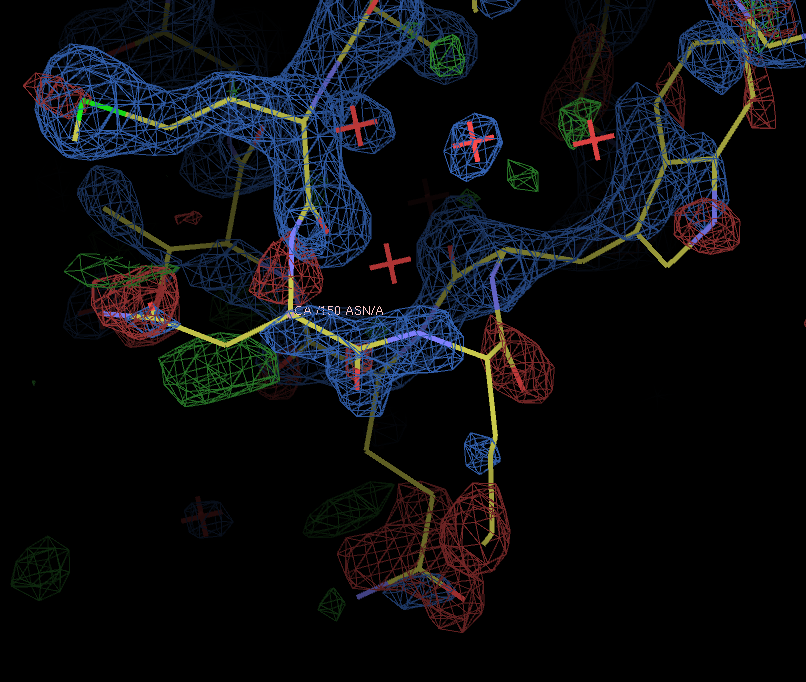


**(c)** 2AGK – residues 150-152 fell within the 2mF_0_-DF_c_ map (blue) but several parts of the model overlapped with unfavorable regions in the mF_0_-DF_c_ map (red), causing us to reject this turn in statistical analysis.

Maps were obtained from the Uppsala University EDS server:

GJ Kleywegt, MR Harris, JY Zou, TC Taylor, A Wählby & TA Jones (2004), "*The Uppsala Electron-Density Server*", Acta Cryst. D60, 2240-2249

Map analysis was conducted in WinCOOT:

Paul Emsley and Kevin Cowtan (2004) “*Coot: Model-Building Tools for Molecular Graphics*” Acta Crystallographica D60,2126-2132
